# Immunosensor with Enhanced Electrochemiluminescence Signal Using Platinum Nanoparticles Confined within Nanochannels for Highly Sensitive Detection of Carcinoembryonic Antigen

**DOI:** 10.3390/molecules28186559

**Published:** 2023-09-11

**Authors:** Huihua Zhang, Chaoyan Zhang, Hui Qu, Fengna Xi

**Affiliations:** 1 Shanxi Bethune Hospital, Shanxi Academy of Medical Sciences, Tongji Shanxi Hospital, Third Hospital of Shanxi Medical University, Taiyuan 030032, China; quhui1986@hotmail.com; 2 Tongji Hospital, Tongji Medical College, Huazhong University of Science and Technology, Wuhan 430030, China; 3 School of Chemistry and Chemical Engineering, Zhejiang Sci-Tech University, Hangzhou 310018, China; 202130107346@mails.zstu.edu.cn

**Keywords:** immunosensor, electrochemiluminescence, confined nanocatalyst, signal enhancement, carcinoembryonic antigen

## Abstract

Rapid, highly sensitive, and accurate detection of tumor biomarkers in serum is of great significance in cancer screening, early diagnosis, and postoperative monitoring. In this study, an electrochemiluminescence (ECL) immunosensing platform was constructed by enhancing the ECL signal through in situ growth of platinum nanoparticles (PtNPs) in a nanochannel array, which can achieve highly sensitive detection of the tumor marker carcinoembryonic antigen (CEA). An inexpensive and readily available indium tin oxide (ITO) glass electrode was used as the supporting electrode, and a layer of amino-functionalized vertically ordered mesoporous silica film (NH_2_-VMSF) was grown on its surface using an electrochemically assisted self-assembly method (EASA). The amino groups within the nanochannels served as anchoring sites for the one-step electrodeposition of PtNPs, taking advantage of the confinement effect of the ultrasmall nanochannels. After the amino groups on the outer surface of NH_2_-VMSF were derivatized with aldehyde groups, specific recognition antibodies were covalently immobilized followed by blocking nonspecific binding sites to create an immunorecognition interface. The PtNPs, acting as nanocatalysts, catalyzed the generation of reactive oxygen species (ROS) with hydrogen peroxide (H_2_O_2_), significantly enhancing the ECL signal of the luminol. The ECL signal exhibited high stability during continuous electrochemical scanning. When the CEA specifically bound to the immunorecognition interface, the resulting immune complexes restricted the diffusion of the ECL emitters and co-reactants towards the electrode, leading to a reduction in the ECL signal. Based on this immune recognition-induced signal-gating effect, the immunosensor enabled ECL detection of CEA with a linear range of 0.1 pg mL^−1^ to 1000 ng mL^−1^ with a low limit of detection (LOD, 0.03 pg mL^−1^). The constructed immunosensor demonstrated excellent selectivity and can achieve CEA detection in serum.

## 1. Introduction

Carcinoembryonic antigen (CEA) is a glycoprotein involved in cell adhesion and is originally extracted from the human colon and embryonic tissues [1,2]. It has been proven that CEA is expressed during fetal development in humans, but its expression levels are suppressed to relatively low levels after birth and during growth. However, in certain malignant tumor patients, especially those with colorectal cancer, CEA can circulate in the bloodstream in high concentrations. Other malignancies, such as melanoma, gastric cancer, breast cancer, ovarian cancer, lung cancer, and pancreatic cancer, may also result in elevated CEA levels. Therefore, CEA serves as an important broad-spectrum tumor biomarker with widespread applications in cancer screening, early diagnosis, and postoperative monitoring [3,4,5]. For example, changes in CEA levels can be used to evaluate the effectiveness of treatment, predict patient prognosis, and monitor cancer recurrence. Therefore, rapid, highly sensitive, and accurate detection of CEA in serum is of great significance.

Immunoassay is the most commonly used strategy for CEA determination that detects the signal changes caused by the binding of CEA and specific antibodies. Depending on the signal, methods including the enzyme-linked immunosorbent assay (ELISA) [6], radioimmunoassay (RIA), chemiluminescence immunoassay (CLIA) [7], electrochemical immunoassay [8,9,10,11], and electrochemiluminescence (ECL) immunoassay [12,13] have been developed. However, an ELISA usually requires a longer detection time and may be influenced by interfering factors. An RIA involves radioactive substances. Chemiluminescence has advantages, such as high sensitivity, a short detection time, and a high level of automation, but it suffers from strict requirements for sample handling and storage. Electrochemical methods offer fast and sensitive detection, but reproducibility remains a challenge. In recent years, ECL technology, also known as electrogenerated chemiluminescence, has gained widespread attention due to its low background, high sensitivity, fast detection speed, and low cost [14,15,16,17,18]. As a technique that combines electrochemistry with chemiluminescence, ECL refers to the generation of luminescent species by inducing high-energy electron transfer reactions on the electrode surface through applied voltage. Until now, a luminol-based ECL system that uses luminol as the luminescent reagent and hydrogen peroxide (H_2_O_2_) as a co-reactant has been widely used in fields such as bioanalysis, environmental testing, and chemical sensing [19,20]. On the one hand, luminol loses a proton to become the luminol anion in an alkaline environment, then undergoes electrochemical oxidation to form the luminol radical. On the other hand, the electrochemical oxidation of H_2_O_2_ generates reactive oxygen species (ROS). Then, the luminol radical reacts with ROS to reach the excited state and emits light when it returns to the ground state. Promoting the generation of ROS during the ECL process is effective at enhancing the sensitivity of the luminol-based ECL sensors.

So far, various materials have been used as catalysts to enhance the detection performance of the sensing platform [21,22,23,24]. Among them, noble metal nanomaterials have attracted wide attention due to their large surface area, excellent conductivity, and high catalytic activity [20,21,22]. For example, platinum (Pt) nanomaterials have good catalytic activity and surface characteristics, enabling the catalysis of H_2_O_2_ to generate ROS [25,26,27,28,29,30]. Commonly, the surface of Pt nanomaterials contains many active sites where Pt atoms can react with H_2_O_2_ molecules, causing localized oxidation and reduction reactions of oxygen and hydrogen atoms. At the same time, Pt nanomaterials can facilitate the decomposition of H_2_O_2_, thereby forming ROS. However, Pt nanomaterials prepared by traditional chemical synthesis methods tend to aggregate. In situ growth of noble metal nanomaterials within confined nanospaces allows for the production of high-performance nanocatalysts [31,32]. Specifically, confined spaces can restrict the diffusion and aggregation of reactants, allowing reactions to occur in very small areas. Thus, control of the size and morphology of noble metal nanomaterials can be achieved in a more uniform nucleation and growth process. In addition, due to the higher surface area-to-volume ratio provided by confined spaces, in situ synthesized noble metal nanomaterials have more active sites and surface reactivity, thereby enhancing their catalytic activity. Therefore, in situ preparation of noble metal nanocatalysts within confined spaces provides new possibilities for the fabrication of highly sensitive ECL sensors.

The growth of vertically ordered mesoporous silica nanochannel film (VMSF) on the electrode surface is an important approach for constructing nanostructured electrodes [33,34,35,36]. Currently, large-area VMSF-modified electrodes (e.g., tens of square centimeters) can be conveniently prepared using the Stöber solution growth method [37,38,39,40,41]. Alternatively, electrochemically assisted self-assembly (EASA) can be used to rapidly grow VMSF on the electrode surface within 5–30 s [42,43,44]. VMSF possesses an array of ultra-small nanochannels perpendicular to the substrate electrode with channel diameters typically in the range of 2–3 nanometers and a film thickness adjustable between 20–200 nanometers [45,46]. The high density of the nanochannels can reach up to 75,000 per square micrometer [47,48,49]. Thus, VMSF has a robust and three-dimensional (3D) silica framework with the nanochannels integrated with the external surface. This unique structure of VMSF confers several advantages to the modified electrode. On the one hand, the VMSF modification layer exhibits minimal swelling during use, greatly enhancing the stability and reproducibility of the modified electrode [50,51]. Furthermore, VMSF includes two independent modification regions, including the nanochannel array and the outer surface. This makes it easier to integrate multifunctionality into the electrode. For example, the nanochannel array of VMSF can serve as a carrier for immobilizing a large amount of stable nanocatalysts, while the outer surface of VMSF can be used to immobilize antibodies or other biorecognition ligands. By spatially separating the processes of immunorecognition and ECL emission, functional integration is achieved under the diffusion of small molecules in the external solution. Based on the 3D structure of VMSF, the integration of in situ grown nanocatalysts and immunorecognition interfaces holds promise for the preparation of high-performance ECL immunosensors.

In this study, an ECL immunosensing platform for the highly sensitive detection of CEA was constructed by functionalizing nanochannels and the outer surface of VMSF. Amino-functionalized VMSF (NH_2_-VMSF) was rapidly grown on an inexpensive and readily available indium tin oxide (ITO) electrode using an electrochemically assisted self-assembly method (EASA). The amino groups inside the nanochannels served as anchoring sites for the in situ electrodeposition of the platinum nanoparticles (PtNPs) via one-step electrodeposition, while the amino groups on the outer surface were applied for the covalent immobilization of recognition antibodies after aldehyde derivatization, forming an immunorecognition interface. Due to the catalytic activity of PtNPs in decomposing H_2_O_2_ to generate ROS, the ECL signal of the luminol–H_2_O_2_ system was significantly enhanced and exhibited high stability. When CEA was present, its binding on the immunorecognition interface formed large-sized immunocomplexes, which hindered the diffusion of the luminescent species and co-reactant to the underlying electrode surface, resulting in a decrease in the ECL signal. Based on the signal-gating effect induced by the immunorecognition, the detection of CEA with high sensitivity could be achieved.

## 2. Results and Discussion

### 2.1. Construction of Immunosensors and Signal-Gated ECL Detection

**Figure 1:**
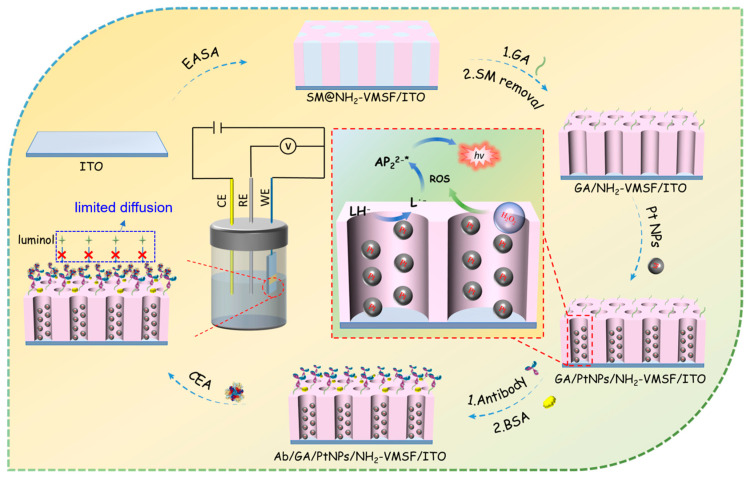
Schematic illustration of the fabrication of the immunosensor and CEA detection based on immunorecognition-induced signal gating phenomenon.

Figure 1 is the schematic illustration of the fabrication of the immunosensor and CEA detection. As shown, the indium tin oxide (ITO) electrode was used as the supporting electrode for the immunosensor fabrication. As a transparent conductive material widely used in modern electronic devices, the ITO electrode exhibits good conductivity, low resistance, excellent corrosion resistance, and stability. Additionally, ITO electrodes are suitable for patterning, making them highly promising for low-cost, disposable sensor devices. As demonstrated, a layer of NH_2_-VMSF was grown on the clean ITO surface using the traditional electrochemical-assisted self-assembly (EASA) method. This method combines electrochemical deposition and self-assembly techniques by applying a negative potential to the electrode, inducing a pH gradient on the electrode surface through reducing hydrogen ions. This gradient facilitates the self-assembly of surfactant micelles (SMs) and the sol–gel process of siloxane precursors on the electrode surface. The resulting film is enriched with amino groups due to the use of amino-functionalized siloxane precursors and contains surfactant micelles within the formed pores (SM@ NH_2_-VMSF/ITO). The surfactant micelles can be easily removed by immersing the SM@ NH_2_-VMSF/ITO in a hydrochloric acid–ethanol solution, yielding an array of open nanochannels. To immobilize the antibodies (Abs) on the outer surface of the VMSF, the amino groups were modified using glutaraldehyde (GA), which generated aldehyde groups for covalent antibody immobilization. To prevent GA from entering the nanochannels and affecting the permeability, a strategy involving GA derivatization followed by SM removal was employed to ensure that aldehyde derivatization occurred only on the outer surface of the NH_2_-VMSF. Subsequently, PtNPs were electro-deposited in situ within the nanochannels. The amino sites within the nanochannels served as anchoring points for the growth of PtNPs, improving their stability. Finally, CEA antibodies were covalently immobilized on the aldehyde-functionalized surface, and after blocking nonspecific sites with BSA, the immunosensor was obtained (Ab/GA/PtNPs@NH_2_-VMSF/ITO). It can be observed that in the 3D structure of the NH_2_-VMSF, the outer surface and nanochannels are divided into two independent functional domains. The outer surface serves as the immunorecognition interface, while the PtNPs deposited inside the nanochannels act as nanocatalysts.

For CEA detection, luminol was used as the ECL emitter and H_2_O_2_ was employed as the co-reactant of the luminol, while the PtNPs served as the co-reactant promoter or an effective catalyst for the H_2_O_2_. They facilitated the electrocatalytic generation of a large amount of ROS, which reacts with the luminescent radical generated by the electrochemical oxidation of the luminol, leading to the formation of excited-state luminol and the subsequent emission of light. Thus, the catalytic effect of PtNPs can effectively enhance the ECL signal intensity in the luminol–H_2_O_2_ system, thereby improving the detection sensitivity. When CEA is present, antibodies on the immunorecognition interface can selectively capture CEA, forming an immunocomplex. The spatial hindrance effect caused by the immunocomplex reduces the diffusion of luminol and H_2_O_2_ toward the surface of the electrode, thus decreasing the ECL signal. Based on this immunorecognition-induced signal-gating phenomenon, ECL detection of CEA can be achieved.

### 2.2. Characterization of NH_2_-VMSF

**Figure 2:**
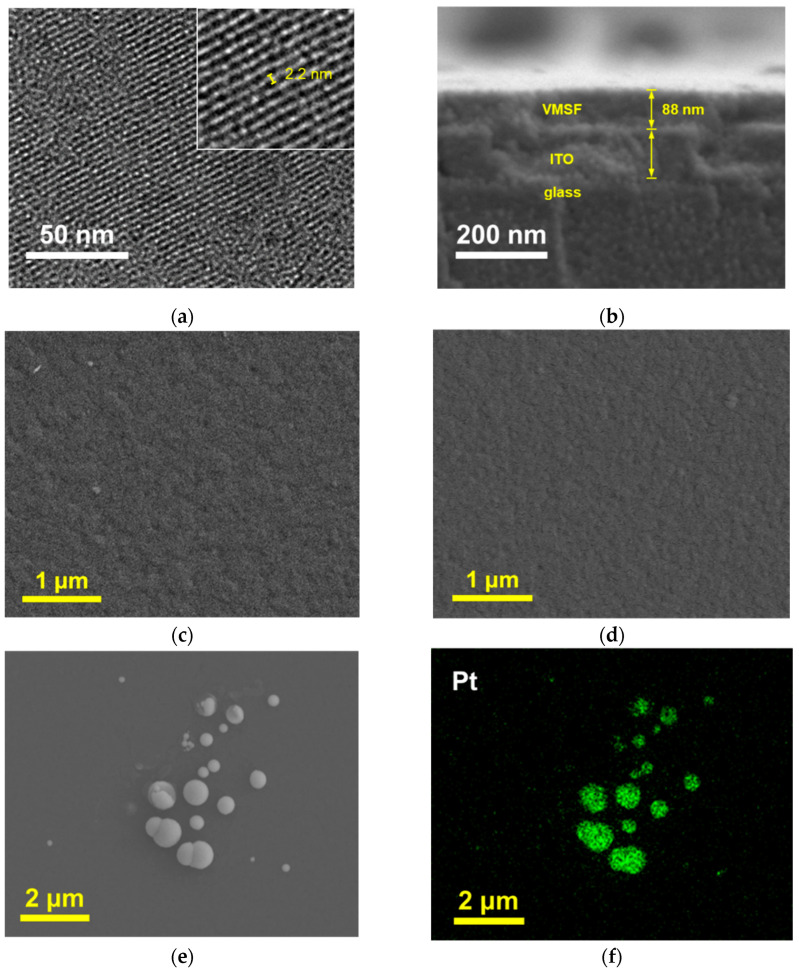
(**a**) Top-view TEM image of NH_2_-VMSF. Inset is the high-resolution TEM image. (**b**) Cross-sectional SEM image of NH_2_-VMSF/ITO. (**c,d**) Top-view of SEM image of NH_2_-VMSF/ITO (**c**) and PtNPs@NH_2_-VMSF/ITO (**d**). (**e**,**f**) SEM image (**e**) and elemental mapping (**f**) of the electrode obtained after NH_2_-VMSF on PtNPs@NH_2_-VMSF/ITO was removed by dissolution with NaOH solution.

TEM and SEM were used to characterize the morphology of NH_2_-VMSF/ITO. The top-view TEM image in Figure 2a reveals that the nanopores are well-ordered and arranged in a hexagonal array. No cracks or fractures are observed within the observed area. The pore size is uniform, measuring approximately 2–3 nm. The cross-sectional SEM image of the VMSF/ITO electrode was also investigated. Due to the non-conductivity of both the glass and VMSF layers, a gold spraying treatment is required before SEM characterization, resulting in the indistinct density difference among the glass, ITO, and VMSF layers. However, the SEM image in Figure 2b still reveals the three-layer structure of the VMSF, ITO, and glass from top to bottom. The thickness of NH_2_-VMSF is approximately 88 nm.

### 2.3. Characterization of PtNPs@NH_2_-VMSF

In order to verify the successful modification of the PtNPs inside the nanochannels, SEM was used to characterize different electrodes. As shown in Figure 2c, the NH_2_-VMSF/ITO electrode without PtNP deposition exhibits a flat surface. Upon PtNP modification, there was no significant change in the surface morphology of the electrode, indicating an absence of large nanostructures formed on the outer surface of NH_2_-VMSF (Figure 2d). To further confirm this, the NH_2_-VMSF layer on the PtNPs@NH_2_-VMSF/ITO electrode was dissolved and removed using NaOH, followed by SEM characterization of the resulting electrode surface. As shown in Figure 2e, numerous spherical structures of varying sizes appear on the ITO substrate, and elemental mapping confirms that these spherical structures correspond to Pt materials (Figure 2f). This can be attributed to the fact that, when NH_2_-VMSF/ITO is dissolved, PtNPs lose their protection from the nanochannels and undergo aggregation, thus confirming that the electrodeposited PtNPs are confined within the nanochannels.

**Figure 3:**
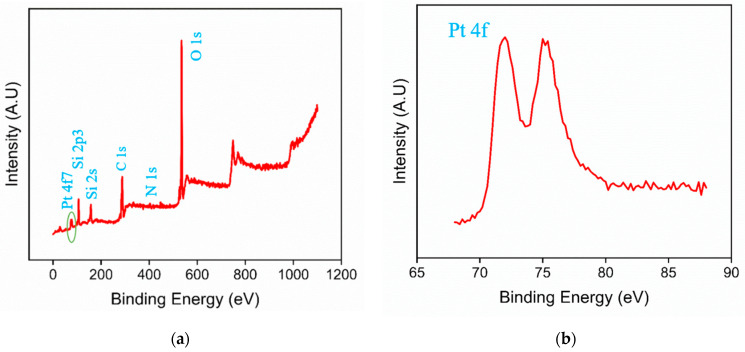
XPS survey spectrum (**a**) and high-resolution Pt_4f_ spectrum (**b**) of PtNPs@NH_2_-VMSF/ITO.

XPS analysis was further conducted on the PtNPs@NH_2_-VMSF/ITO electrode to determine its elemental composition. Figure 3a shows the XPS survey spectrum, which reveals significant peaks corresponding to O, Si, Pt, and N elements. Figure 3b presents the high-resolution Pt_4f_ spectrum with two strong peaks observed at around 71.5 eV and 75.4 eV, confirming the presence of platinum. The results confirm the presence of amino-functionalized silica structures and deposited Pt nanoparticles in the PtNPs@NH_2_-VMSF modification layer.

### 2.4. The Enhanced ECL by PtNPs

**Figure 4:**
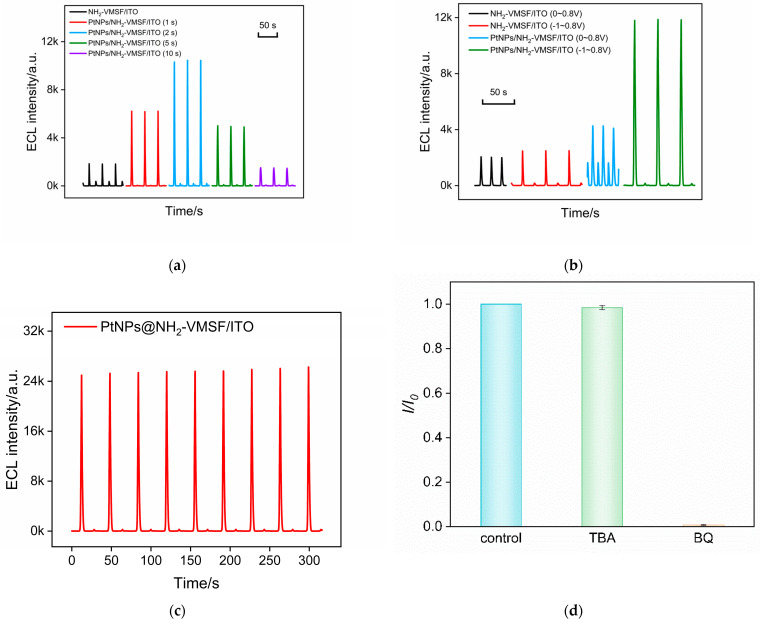
(**a**) ECL signal obtained on PtNPs/NH_2_-VMSF/ITO electrode prepared using different deposition times for the in situ synthesis of PtNPs. (**b**) ECL signal obtained on PtNPs/NH_2_-VMSF/ITO electrode under different potential ranges. (**c**) ECL signal under continuous CV scanning. (**d**) The relative ratio of ECL intensity in absence (*I*_0_) or presence (*I*) of different free radical scavengers.

The influence of PtNP deposition time on the ECL performance of the electrode was examined. As shown in Figure 4a, the ECL intensities of PtNPs@NH_2_-VMSF/ITO electrodes obtained with different deposition times were compared. The results reveal that the ECL intensity of the electrode initially increases and then decreases with increasing PtNP deposition time. This may be attributed to the fact that a longer deposition time can increase the amount of deposited PtNPs, but an excessively large nanoparticle size due to prolonged deposition time could decrease the diffusion of ECL emitters and co-reactants inside the nanochannels, resulting in reduced ECL intensity. To achieve the highest ECL signal, the electrode with a PtNP deposition time of 2 s was selected for further experiments. Compared to NH_2_-VMSF/ITO electrodes without nanocatalysts, the ECL response measured on PtNPs@NH_2_-VMSF/ITO electrodes was enhanced four-fold.

ECL measurements at different scan potential ranges were further conducted on the electrodes before and after PtNP deposition to further validate the catalytic effect of PtNPs. When the scan potential range was set from 0 V to 0.8 V, the electrode with PtNP deposition exhibited a certain improvement in ECL signal compared to the unmodified NH_2_-VMSF/ITO electrode (Figure 4b). However, the enhancement of the ECL signal was not significant. On the contrary, when the initial scan potential was shifted to −1 V, the ECL signal of the PtNPs@NH_2_-VMSF/ITO electrode showed a remarkable increase. This demonstrates that the significant enhancement in the ECL signal is attributed to the electrocatalysis of H_2_O_2_ by the PtNPs at negative potentials.

To examine the stability of the ECL signal, the electrode was subjected to continuous electrochemical scans, and its ECL signal was recorded. As shown in Figure 4c, there was no significant change in the ECL signal of the PtNPs@NH_2_-VMSF/ITO electrode, demonstrating high stability.

In order to verify the sensitization mechanism of PtNPs on the ECL of the luminol–H_2_O_2_ system, the possible reactive oxygen species (ROS) were investigated by adding various radical scavengers. As shown in Figure 4d, when the superoxide anion (O^2•−^) scavenger benzoquinone (BQ) was added, the ECL signal significantly decreased. However, when the hydroxyl radical (·OH) scavenger tert-butanol (TBA) was present, the ECL signal of the system remained relatively constant. This indicates that the ROS involved in this ECL process is O^2−^. A possible ECL mechanism during a CV scan in the presence of nanocatalysis is illustrated in the following equations. Briefly, luminol loses a proton to form the luminate anion (LH^−^), which then loses an electron during the electrochemical oxidation process to generate the luminol radical (L^•−^). H_2_O_2_ is electrochemically catalyzed by PtNPs to produce a large amount of O^2•−^, which reacts with L^•−^ to form the excited state of 3-aminophthalate (AP^2−∗^). The emission of ECL occurs during the transition of the excited state back to the ground state (AP^2−^).

$${LH}^{-}-e^{-}\to L^{•-}+H^{+}$$

$$H_{2}O_{2}-e^{-}\overset{PtNPs}{\to }O_{2}^{•-}$$

$$L^{•-}+O_{2}^{•-}\to {AP}^{2-*}+O_{2}$$

$${AP}^{2-*}\to {AP}^{2-}+hv$$

### 2.5. Feasibility of Immunosensor Fabrication and Optimization of CEA Detection Conditions

**Figure 5:**
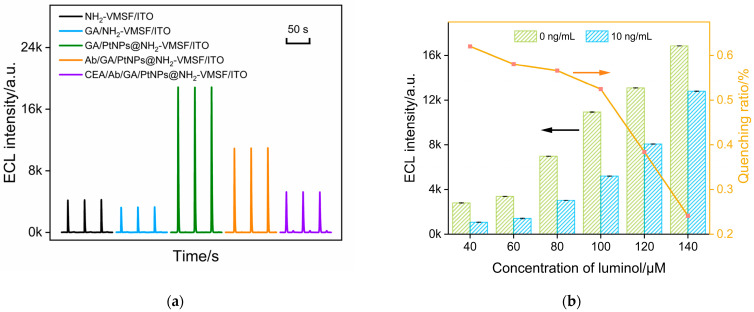

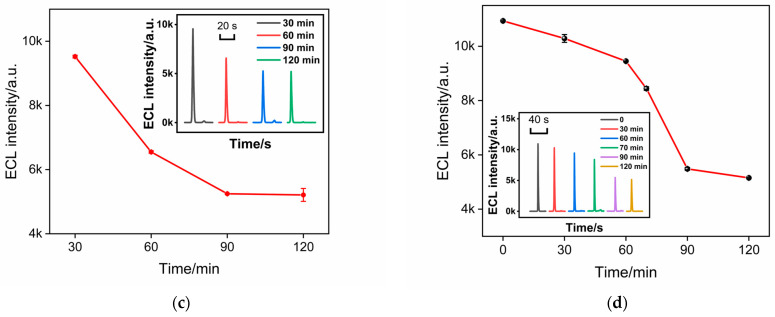
(**a**) ECL signal on different electrodes obtained in the fabrication of the immunosensor. (**b**) The ECL intensity and the proportion of ECL signal reduction caused by the gate effect in the luminol systems of different concentrations before and after adding CEA. (**c**) The ECL signal obtained after CEA binds with the immunosensor prepared using different incubation times for Ab immobilization. Inset is the corresponding ECL intensity. (**d**) The ECL intensity of the immunosensor obtained using different incubation times for CEA binding. Inset is the corresponding ECL intensity.

The feasibility of immunosensor fabrication was examined by investigating the ECL signals of the obtained electrodes during the construction process. As shown in Figure 5a, the ECL signal of the luminol–H_2_O_2_ system on the NH_2_-VMSF/ITO electrode is low. The modification of GA on the outer surface of SM@NH_2_-VMSF and the following removal of SMs did not affect the ECL signal of the electrode. However, after the deposition of PtNPs inside the nanochannels, the ECL signal on the GA/PtNPs@NH_2_-VMSF/ITO electrode was significantly enhanced. This enhancement is attributed to the electrocatalytic production of ROS by PtNPs towards H_2_O_2_ at negative potentials, thereby enhancing the ECL of the luminol. The covalent immobilization of Abs and the blocking of nonspecific sites by BSA resulted in a decrease in the ECL signal of the fabricated immunosensor (Ab/GA/PtNPs@NH_2_-VMSF/ITO). Clearly, when Abs and the blocking BSA are immobilized on the outer surface of NH_2_-VMSF, they hinder the diffusion of luminol and H_2_O_2_. In the presence of CEA in the system, the ECL signal of the electrode further decreases. This reduction is attributed to the specific recognition of CEA by the immunorecognition interface. As the immunocomplexes formed on the outer surface of NH_2_-VMSF, also on the exit of the nanochannels, they significantly hindered the diffusion of small molecules to the electrode surface, resulting in decreased ECL intensity.

The concentration of ECL luminescent emitter is one of the factors influencing the gated signal. Thus, the effect of luminol concentration on CEA detection was investigated. Figure 5b shows the proportion of ECL signal reduction caused by the gate effect in the luminol systems of different concentrations before (*I*_0_) and after (*I*) adding CEA. It can be observed that as the concentration of luminol increases, the absolute value of the ECL signal increases, but the quenching rate, calculated using (*I*_0_ − *I)/I*_0_, caused by the gate effect is low. The luminol concentration (100 μM) with the highest ECL quenching rate after CEA binding was selected for further investigation.

**Figure 6:**
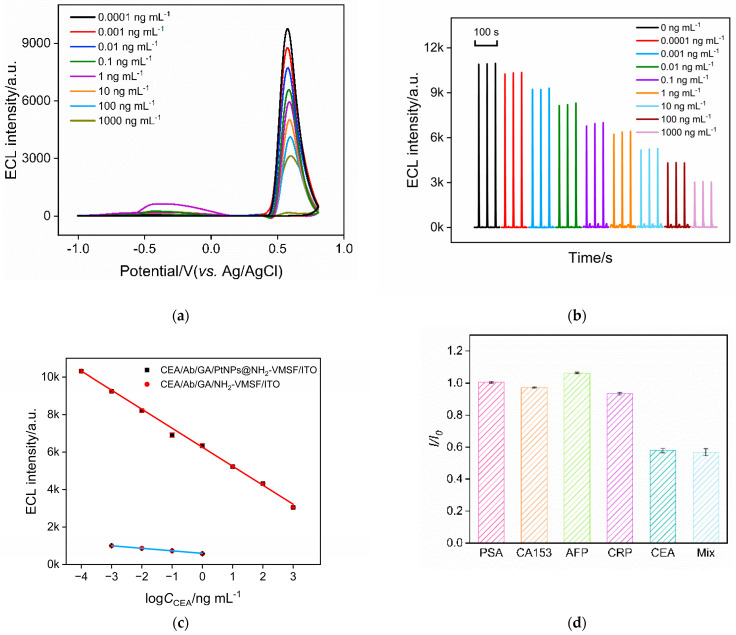
(**a**) ECL–potential plots obtained on Ab/GA/PtNPs@NH_2_-VMSF/ITO electrode in presence of different concentrations of CEA. (**b**) ECL signal obtained on the fabricated immunosensor after incubation with different concentrations of CEA. (**c**) The linear fitting curve for the detection of CEA using Ab/GA/PtNPs@NH_2_-VMSF/ITO (the line above) or Ab/GA/NH_2_-VMSF/ITO electrode (the line below). (**d**) The change ratio of ECL signal in absence (*I*_0_) or presence (*I*) of different species. The concentration of PSA, AFP, CRP, CEA is 1 ng mL^−1^, and the concentration of CA15-3 is 1 mU mL^−1^.

To achieve the highest sensitivity for the detection of CEA, the influence of antibody incubation time and CEA binding time on the performance of the immunosensor was also investigated. The ECL signal after the binding of CEA with immunosensors fabricated using different Ab immobilization times was investigated. As shown in Figure 6c, the ECL signal significantly decreased with the increase in the incubation time. After the antibody incubation time reached 90 min, the ECL signal reached a plateau. Thus, the antibody incubation time was chosen as 90 min in the subsequent experiments. In addition, when the CEA binding time with the immunorecognition interface reached 90 min, the ECL signal was the lowest. Thus, the binding time was set at 90 min.

### 2.6. ECL Determination of H_2_O_2_

Under optimal detection conditions, the performance of the constructed immune sensor for detecting CEA was investigated. Figure 6a shows the ECL–potential plots obtained on the Ab/GA/PtNPs@NH_2_-VMSF/ITO electrodes in the presence of different concentrations of CEA. The ECL signal of the electrode after incubation with different concentrations of CEA is demonstrated in Figure 6b. As the CEA concentration increases, the number of immune complexes formed on the electrode surface increases. This leads to a stronger hindrance to the diffusion of the ECL emitters and co-reactants, resulting in a further decrease in the ECL signal of the electrode. Consequently, quantitative detection of CEA is achieved. Figure 6c shows the corresponding linear fitting curve. The results indicate a good linear relationship between the ECL signal (I_ECL_) and the logarithm of CEA concentration (logC_CEA_) in the range of 0.1 pg mL^−1^ to 1000 ng mL^−1^ (I_ECL_ = −1015 logC_CEA_ + 6261, R^2^ = 0.996). The limit of detection (LOD), calculated based on a signal-to-noise ratio (S/N) of 3, was determined to be 0.3 pg mL^−1^.

As a comparison, the performance of the immunosensor constructed using an NH_2_-VMSF/ITO electrode for detecting CEA was also investigated. As shown in Figure 6c, the linear range for CEA detection is from 1 pg mL^−1^ to 1 ng mL^−1^ (I_ECL_ = −135logC_CEA_ + 595, R^2^ = 0.999). It is evident that the introduction of PtNPs as nanocatalysts enhances the ECL signal and significantly improves the detection linear range and sensitivity of the immunosensor.

### 2.7. Selectivity and Real Sample Analysis

The common tumor biomarkers, including prostate specific antigen (PSA), carbohydrate antigen 15-3 (CA153), alpha-fetoprotein (AFP), and C-reactive protein (CRP), were selected as potential interferents to investigate the selectivity of the immunosensor. As shown in Figure 6d, these tumor biomarkers did not cause a significant change in the ECL signal of the immunosensor except when CEA was present. This indicates that the prepared immunosensor exhibits excellent selectivity.

The reliability of the fabricated immunosensor was assessed by detecting CEA in real serum samples. Five human serum samples (healthy, female) were detected. The concentrations of CEA detected by the constructed immunosensor were found to be 1.87 ± 0.03 ng mL^−1^, 0.69 ± 0.02 ng mL^−1^, 2.36 ± 0.07 ng mL^−1^, 1.10 ± 0.03 ng mL^−1^, 2.88 ± 0.10 ng mL^−1^ (mean ± SD, n = 3), and they closely match the results (1.95 ± 0.04 ng mL^−1^, 0.77 ± 0.02 ng mL^−1^, 2.28 ± 0.06 ng mL^−1^, 1.17 ± 0.04 ng mL^−1^, 2.83 ± 0.09 ng mL^−1^) obtained using the commercially used ECL analyzer (Cobas 6000, Roche, Basel, Switzerland). In addition, the accuracy of the detection was evaluated using a standard addition method. Commonly, the CEA concentration of a healthy person is less than 5 ng mL^−1^. Different concentrations of CEA were added to the serum to simulate the blood samples of cancer patients (> 5 ng mL^−1^). As shown in Table 1, the recovery rate of the detection ranged from 98.7% to 107%, and the relative standard deviation (RSD) of the determination was no higher than 3.2%, demonstrating the accuracy of the determination.

## 3. Materials and Methods

### 3.1. Chemicals and Materials

Hexahydrate chloroplatinic acid (H_2_PtCl_6_·6H_2_O), luminol, sodium dihydrogen phosphate dihydrate (NaH_2_PO_4_·2H_2_O), disodium hydrogen phosphate dodecahydrate (Na_2_HPO_4_·12H_2_O), tetraethoxysilane (TEOS, 98%), cetyltrimethylammonium bromide (CTAB), 3-aminopropyltriethoxysilane (APTES), sodium hydroxide (NaOH), bovine serum albumin (BSA) were purchased from Shanghai Aladdin Biochemical Technology Co., Ltd. (Shanghai, China). Sulfuric acid, acetone, anhydrous ethanol (99.8%), and concentrated hydrochloric acid (HCl, 38%) were obtained from Hangzhou Shuanglin Reagent Co., Ltd. (Hangzhou, China). Carcinoembryonic antigen (CEA), anti-CEA antibody, alpha-fetoprotein (AFP), and cancer antigen 15-3 (CA 15-3) were purchased from Beijing Key Laboratory Biotechnologies Co., Ltd. (Beijing, China). C-reactive protein (CRP) was purchased from Nanjing OE Biotech Co., Ltd. (Nanjing, China). Prostate-specific antigen (PSA) was purchased from Beijing Boko Biotechnology Co., Ltd. (Beijing, China). Phosphate-buffered saline (PBS, 0.01 M, pH = 7.4) was prepared by mixing sodium dihydrogen phosphate and disodium hydrogen phosphate in certain proportions. All antigen samples were gradiently diluted in PBS (0.01 M, pH = 7.4). All chemical reagents used were of analytical grade. Ultrapure water (18.2 MΩ·cm) was used for the preparation of all solutions during the experiment. Indium tin oxide (ITO) conductive glass (square resistance < 17 Ω/sq, ITO thickness: 100 ± 20 nm) was purchased from Zhuhai Kaiwei Optoelectronics Technology Co., Ltd. (Zhuhai, China).

### 3.2. Characterizations and Instrumentations

Transmission electron microscopy (TEM, Hitachi HT7700) and scanning electron microscopy (SEM, Zeiss ULTRA 55) were used to characterize the morphology of VMSF and PtNPs. For TEM sample preparation, the VMSF layer was carefully scraped off the electrode using a knife and dispersed in anhydrous ethanol for ultrasonic dispersion. The resulting dispersion was drop-casted onto a copper grid. After being air dried, the sample was characterized at an accelerating voltage of 200 kV. For SEM sample preparation, NaOH (2 M, 50 μL) was drop-casted onto the surface of PtNPs@VMSF/ITO to etch the VMSF. After reaction for 5 min, NaOH on the surface was slowly rinsed off with ultrapure water, and the resulting material was left to air dry. Cyclic voltammetry (CV) testing was performed on an electrochemical workstation (CHI660D, Shanghai Chenhua, Shanghai, China). X-ray photoelectron spectroscopy (XPS) analysis was conducted using a PHI5300 electron spectrometer (PE Ltd., Cincinnati, OH, USA) with Mg Kα radiation source (250 W, 14 kV). All experiments employed a traditional three-electrode system with Ag/AgCl serving as the reference electrode, platinum wire as the counter electrode, and the modified electrode as the working electrode.

### 3.3. Preparation of NH_2_-VMSF/ITO and PtNPs@NH_2_-VMSF/ITO Electrode

The electrochemical-assisted self-assembly (EASA) method reported in the literature was used to grow amino-functionalized vertically ordered mesoporous silica film (NH_2_-VMSF) on the surface of indium tin oxide (ITO) electrode [52,53]. Briefly, ethanol (20 mL), sodium nitrate solution (0.1 M, pH = 2.36, 20 mL), CTAB (1.585 g), and APTES (318 μL) were mixed. After uniform mixing, the pH of the solution was adjusted to 2.97 with HCl, and TEOS (2732 μL) was then added to the solution. Under vigorous stirring, the obtained precursor solution was allowed to react for 2.5 h. After soaking ITO in a sodium hydroxide solution (1 M) for 60 min, it was sequentially washed with acetone, ethanol, and ultrapure water to give it a negatively charged surface. The ITO electrode was then cut, and an insulating adhesive was used to limit the electrode area to 0.5 cm × 1 cm. Using ITO as the working electrode, a constant current (current density of −0.7 mA/cm^2^) was applied for 10 s to achieve rapid growth of NH_2_-VMSF, followed by a quick rinse with a large amount of ultrapure water. The resulting electrode was dried with nitrogen gas and aged overnight at 120 °C to obtain an electrode with surfactant micelles (SMs) within the nanochannels, denoted as (SM@NH_2_-VMSF/ITO). Finally, the SM@NH_2_-VMSF/ITO electrode was immersed in a HCl–ethanol solution (0.1 M) and stirred for 5 min to remove the micelles, resulting in an electrode with open channels, abbreviated as NH_2_-VMSF/ITO electrode.

The PtNPs@NH_2_-VMSF/ITO electrode was prepared by electrodepositing H_2_PtCl_6_ in situ within the nanochannels of NH_2_-VMSF. Briefly, an H_2_PtCl_6_ solution (3.86 mM in 0.1 M H_2_SO_4_) was used as the electrodepositing solution. The NH_2_-VMSF/ITO electrode was subjected to a constant potential of −0.2 V for 2 s, resulting in the growth of PtNPs exclusively within the nanochannels. The electrode with PtNPs confined to the nanochannels was denoted as PtNPs@NH_2_-VMSF/ITO.

### 3.4. Preparation of the Immunosensor

To prepare the immunosensor, PtNPs@NH_2_-VMSF/ITO electrode was used as the substrate electrode. When constructing the immunosensing interface, glutaraldehyde (GA) was used as a cross-linking agent, and the CEA antibody (Ab) was immobilized through covalent cross-linking. To ensure that GA only modified the outer surface of NH_2_-VMSF, the amino groups on the outer surface of NH_2_-VMSF were treated with aldehyde derivatization before removing the SM. Firstly, GA solution (5%, 50 μL) was drop-coated on the surface of SM@NH_2_-VMSF/ITO electrode, followed by incubation at 37 °C in the dark for 30 min. Then, the electrode was thoroughly rinsed and then immersed in HCl–ethanol solution (0.1 M) for 5 min with stirring to remove micelles, resulting in an electrode with open channels, namely GA/NH_2_-VMSF/ITO electrode. Next, PtNPs were electrodeposited inside the nanochannels following the above steps, resulting in GA/PtNPs@NH_2_-VMSF/ITO electrode. Then, the electrode was immersed in Ab solution (100 μg/mL) and incubated at 4 °C for 90 min. After the reaction, the electrode surface was washed with PBS solution (0.01 M, pH = 7.4) to remove unbound antibodies, and the electrode was then immersed in BSA solution (1%, *w*/*w*) at 4 °C for 90 min to block nonspecific binding sites. Finally, after thorough rinsing of the electrode, the immunosensor named BSA/Ab/GA/PtNPs@NH_2_-VMSF/ITO was obtained.

### 3.5. ECL Detection of CEA

The detection performance of the immunosensor was evaluated by detecting different concentrations of CEA. The medium for the binding between the immuno-recognitive interface with CEA was PBS (0.01 M, pH = 7.4). Briefly, the electrode was incubated with different concentrations of CEA at 4 °C for 90 min. After thorough washing to remove unbound CEA, ECL testing was performed. Specifically, the electrolyte solution for detection was PBS (0.01 M, pH = 7.4) containing luminol (100 μM) and H_2_O_2_ (100 μM). Continuous scanning was conducted using the CV method on the working electrode within the potential ranged from −1 V to 0.8 V using a scan rate of 100 mV/s. CV curve and ECL response curve were simultaneously recorded.

## 4. Conclusions

In summary, an ECL immunosensing platform has been developed based on a 3D rigid nanochannel array film for the highly sensitive detection of the tumor marker CEA. The amino-modified VMSF was prepared in a one-step process on inexpensive and readily available ITO electrodes. The nanochannel array and outer surface were utilized as independent functional domains. The amino groups within the nanochannel array served as anchoring groups for the in situ growth of the platinum nanoparticles, which catalyzed hydrogen peroxide to produce ROS, significantly enhancing the ECL signal of the luminol with high stability. The amino groups on the outer surface were used as chemical reaction groups to covalently immobilize the antibodies after aldehyde derivatization, creating an immunorecognition interface. When CEA specifically bound to the immunorecognition interface, the resulting immunocomplexes reduced the diffusion of luminescent species and co-reactants towards the electrode, leading to a gating effect. By combining the specific recognition of antibodies and the enhanced ECL signal using the platinum nanoparticles, the constructed immunosensor achieved highly sensitive detection of CEA. The immunosensor offers advantages, such as inexpensive and readily available electrode substrates, a simple fabrication, an enhanced ECL signal, and selective recognition interfaces, making it highly promising for applications in tumor marker detection and other biological analyses.

## Figures and Tables

**Table 1:** Detection of CEA in serum sample using standard addition method.

Sample	Spiked ^b^ (ng mL^−1^)	Found (ng mL^−1^)	RSD (%, n = 3)	Recovery (%)
	5.00	5.15	2.3	103
	10.0	9.92	0.8	99.2
Serum ^a^	50.0	51.2	2.1	102.4
	100	98.7	1.4	98.7
	500	535	3.2	107

^a^ Samples with added CEA were diluted by a factor of 50 using the electrolyte. ^b^ The concentration of CEA was the added concentration before dilution.

## Data Availability

The data presented in this study are available on request from the corresponding author.
